# Low dose of extracellular vesicles identified that promote recovery after ischemic stroke

**DOI:** 10.1186/s13287-020-01601-1

**Published:** 2020-02-19

**Authors:** Laura Otero-Ortega, Fernando Laso-García, Mari Carmen Gómez-de Frutos, Luke Diekhorst, Arturo Martínez-Arroyo, Elisa Alonso-López, María Laura García-Bermejo, Macarena Rodríguez-Serrano, Mercedes Arrúe-Gonzalo, Exuperio Díez-Tejedor, Blanca Fuentes, María Gutiérrez-Fernández

**Affiliations:** 1 Neuroscience and Cerebrovascular Research Laboratory, Department of Neurology and Stroke Center, La Paz University Hospital, Hospital La Paz Institute for Health Research (IdiPAZ), Autonomous University of Madrid, Paseo de la Castellana 261, 28046 Madrid, Spain; 2 Biomarkers and Therapeutic Targets Unit, Instituto Ramón y Cajal de investigación Sanitaria (IRYCIS), Madrid, Spain

**Keywords:** Brain repair, Extracellular vesicles, Oxygen and glucose deprivation, Subcortical stroke, White matter lesion

## Abstract

**Background:**

Mesenchymal stem cell-derived extracellular vesicles (EVs) are one of the most promising therapeutics in protective and/or regenerative therapy in animal models of stroke using a dose of 100 μg. However, whether EVs dose is related to outcomes is not known. This study aimed to identify the optimal effective dose of EVs from adipose tissue-derived mesenchymal stem cells that promote functional recovery in subcortical stroke.

**Materials and methods:**

For this purpose, various doses of EVs were tested in an in vitro oxygen-glucose deprivation (OGD) model of oligodendrocytes and neuronal ischemia. At least 50 μg of EVs were necessary to induce proliferation and differentiation of oligodendrocyte and neurons in OGD conditions. For in vivo study, rats were subjected to subcortical stroke and various doses (50 μg, 100 μg, or 200 μg) of EVs were intravenously administered after 24 h.

**Results:**

All the animals in the EV groups showed significant improvement in functional tests, with an increase in tract connectivity and brain repair-associated markers, and a decrease in cell death and in astrocyte-marker expression. Cell proliferation was increased in the groups receiving 50 μg and 100 μg doses. Only the 50-μg dose was associated with significant increases in brain-derived neurotrophic factor expression.

**Conclusion:**

In conclusion, 50 μg of EVs appears to be the minimal effective dose to enhance protection, brain repair, and recovery in subcortical ischemic stroke.

## Background

Stroke is the leading cause of death and disability worldwide [1]. The pathophysiological responses after ischemic stroke are complex, and there is currently no therapy to repair the damage caused after the initial insult. Only intravenous thrombolysis with tissue plasminogen activator (tPA) and endovascular treatment are effective therapies to treat the acute phase [2]. However, these approaches are limited by a narrow therapeutic window (< 4.5 h for tPA and 6–24 h for endovascular treatment) [3, 4]. In this sense, mesenchymal stem cell (MSC) treatment is emerging as a promising therapy due to its post-stroke reparative properties. MSCs participate in processes such as neurogenesis, synaptogenesis, oligodendrogenesis, axonal connectivity, and myelin formation and have been shown to be effective independently of whether the lesion involves gray or white matter [5–7]. The mechanisms underlying these therapeutic effects of stem cells are unknown, but they might be related to paracrine actions through secretory factors in the extracellular space that modify cell behavior favoring brain recovery. In this sense, extracellular vesicles (EVs) have complex functions in cell-to-cell communication and compound exchange, although their physiological roles are still unknown. EVs are vesicles of endosomal origin that carry bioactive molecules (proteins, RNA, DNA, and lipids) and are secreted by all cell types [8, 9]. Due to their ability to cross the blood-brain barrier (BBB), EVs have emerged as novel therapeutic effectors in regenerative medicine for neurological diseases [10, 11]. In particular, EVs derived from MSCs have been shown to promote functional recovery [12–16], providing long-term brain protection [15], gray matter repair, and recovery, and have been associated with an increase in neurogenesis and angiogenesis [14], significant enhancement of white matter repair [13], and immunomodulation [12, 15] in experimental animal models of stroke [17] using a dose of 100 μg [13–16]. The administration of EVs derived from MSCs is still an incipient therapy, and many aspects remain to be resolved. The time of administration, the most effective route, and the minimum effective dose are aspects that still need to be determined before implementing the treatment at the clinical level. In the preclinical studies, there is strong evidence of the importance of dose in neurological outcomes after brain ischemia. Since preclinical data indicate that dose is an important factor in optimizing therapy [18], this raises the question of whether a higher dose of EVs will produce a greater effect in recovery. Therefore, in this study, the aim was to conduct a dose-response study of EVs from adipose tissue-derived MSCs to identify the optimal effective dose to enhance protection, repair, and recovery using an in vitro oxygen and glucose deprivation (OGD) model and in an in vivo model of subcortical stroke in rats.

## Materials and methods

### Ethics statement

In this study, animal care and experimental procedures were strictly in accordance with the Guide for the Care and Use of Laboratory Animals, and the study was approved by La Paz University Hospital’s Ethics Committee for the Care and Use of Animals in Research (Ref. PROEX 361/15), according to the Spanish and European Union rules (86/609/CEE and RD53/2013). The experiments were conducted according to stroke therapy academic industry roundtable [19, 20] and ARRIVE [21] guidelines in terms of randomization, blinding, and statistical power (https://www.nc3rs.org.uk/arrive-guidelines).

### Cell culture protocol, EV isolation and characterization

MSCs obtained from allogeneic adipose tissue of Sprague-Dawley rats (250–300 g) were cultured. The adipose tissue was digested with collagenase (Sigma-Aldrich) and incubated at 37 °C in 5% CO_2_. On the third pass, MSCs were cultured overnight with an EV-free fetal bovine serum culture medium. After 24 h, the cell supernatants were collected and the EVs were obtained using an EV isolation kit (Exoquick, SBI), following the manufacturer’s instructions. For characterization and further administration, the purified EVs were eluted in phosphate-buffered saline (PBS) and stored at − 20 °C until use. The EVs were quantified by measuring the total protein concentration using a bicinchoninic acid commercial kit (Thermo Scientific, Waltham, MA, USA).

**Fig. 1:**
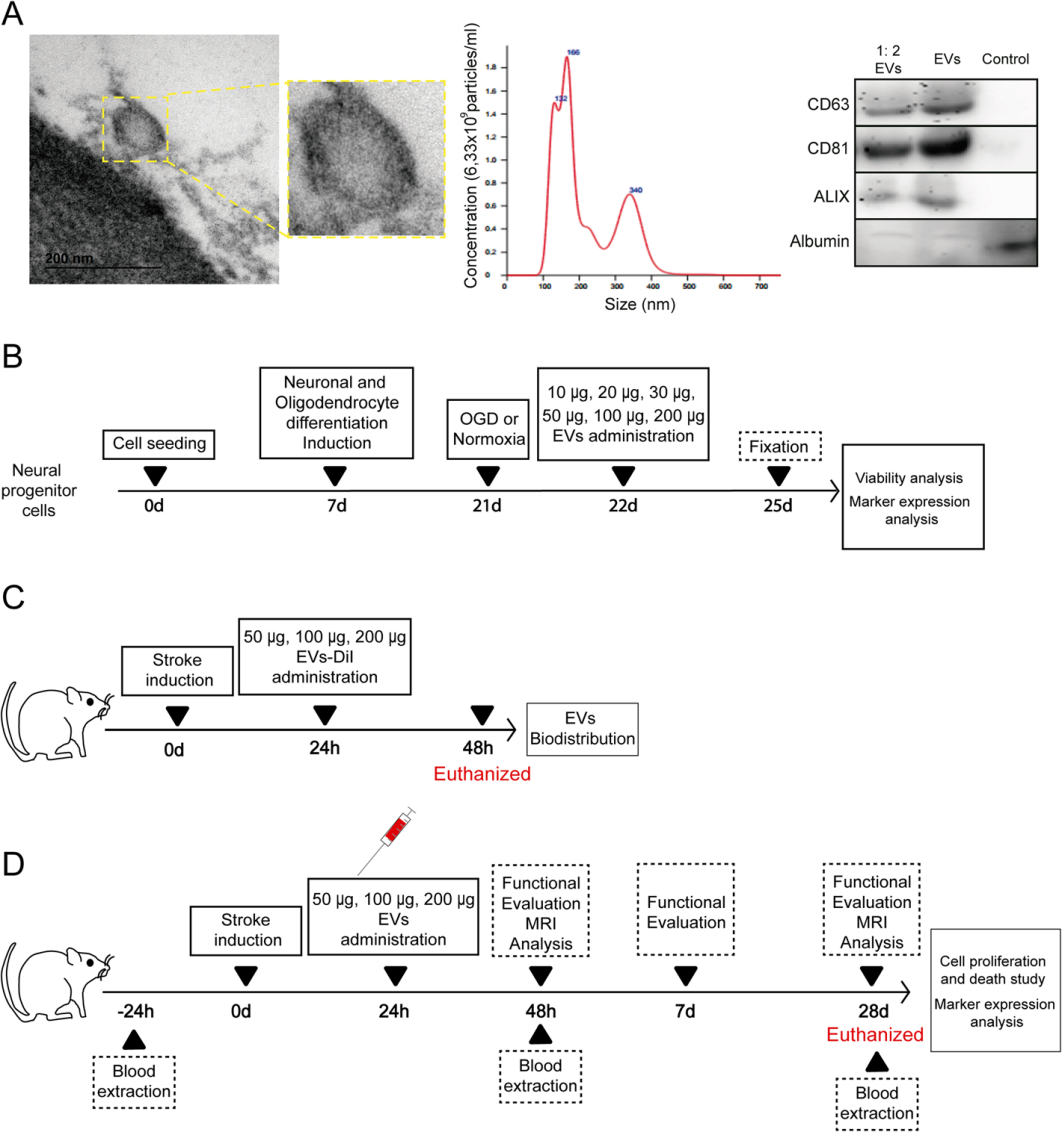
Experimental protocol schematic. **a** EVs characterization using various techniques: EVs imaged by electronic microscope (left); histogram representing a distribution graph of size and concentration of the particles of the EVs using NanoSight (middle); and CD63, CD81, and ALIX as positive markers and albumin as negative marker expression using western blot (right). Supernatant has been used as control. **b** In vitro study. Human neural progenitor cells were plated and cultured, using a seeding density of 1 × 10^4^ cells/cm^2^ and grown to 70% confluence. At 7 days, the cells were differentiated to oligodendrocytes and neurons, with medium changes every 2–3 days for 14 days. At 21 days, cells were subjected to normoxia or OGD and the following day the cells received various doses of EVs (10 μg, 20 μg, 30 μg, 50 μg, 100 μg, or 200 μg) for 72 h. After fixing, proliferation and marker expression were analyzed. **c** Biodistribution study of EVs. Rats were subjected to subcortical infarct by endothelin-1 injection. Twenty-four hours later, EVs were labeled with DiI prior to administration and the rats received treatment (50 μg, 100 μg, or 200 μg of EVs). At 48 h, histological studies for biodistribution of EVs were performed. **d** In vivo study. Subcortical stroke was induced using endothelin-1. Each group received one of various doses of MSC-derived EVs or a saline solution as treatment 24 h after surgery. Functional deficit and MRI scans were evaluated at 48 h and at 28 days after surgery. The blood levels of EVs were analyzed 24 h prior to surgery, and at 48 h and 28 days after surgery. The histological and molecular analyses were performed at 28 days. Abbreviations: EVs, extracellular vesicles; MRI, magnetic resonance image; OGD, oxygen and glucose deprivation

EVs were characterized based on their morphology and size (< 150 nm), using electron microscopy, NanoSight (NanoSight Ltd., UK), and western blot technique. For electron microscopy, the EVs were fixated in 2.5% gluteraldehyde 0.1 M sodium cacodylate solution for 1 h at 4 °C. Post fixation took place using 2% osmium tetroxide for 1 h at 4 °C. The EV pellet was dehydrated and then embedded in resin. Sixty-nanometer-thick sections were cut and observed under transmission electron microscopy at 80 kV. For western blot technique, the EVs were characterized using their phenotype expression of CD63 (1:1000, Abcam), CD81 (1:1000, Abcam), and ALIX (1:1000, Cell Signal) as a positive marker and albumin (11,000, Abcam) as a negative marker (Fig. 1a).

### In vitro assay

The therapeutic effects of various doses of EVs derived from adipose tissue MSCs on oligodendrocytes and neurons subjected to OGD conditions or normal oxygen levels were evaluated.

Human neural progenitor cells were acquired from Sigma-Merck Millipore. Cells were expanded (using a seeding density of 1 × 10^4^ cells/cm^2^) on tissue culture flasks (Fisher Scientific) coated with 20 mg/mL Matrigel (Sigma-Merck Millipore) in human expansion media (Sigma-Merck Millipore) supplemented with neural supplement 1 (× 50), recombinant human basic fibroblast growth factor (bFGF) (50 μg/mL), expansion supplement A (platelet-derived growth factor [PDGF]-AA), and expansion supplement B (NT3). Cells were routinely grown to 70% confluence and were then detached using Accutase (Sigma-Merck Millipore).

For differentiation of human neural progenitor cells into oligodendrocytes and neurons, the progenitor cells were cultured using a seeding density of 1 × 10^4^ cells/cm^2^ at P1 plated on glass coverslips (12 mm circles) with 100 μg/mL poly-L-ornithine and 10 μg/mL laminin into 24-well plates with expansion medium (human expansion media, supplemented with neural supplement 1 (× 50), recombinant human bFGF (50 μg/mL), PDGF-AA, and NT3, until 50% confluence was achieved. Seven days after seeding, the cells were treated with fresh human spontaneous differentiation complete medium supplemented with neural supplement 1 (× 50) for 14 days, with medium changes being done every 2–3 days.

For an in vitro model of ischemia, oligodendrocyte and neurons were plated to reach 70% confluence in a normal medium. At 7 days, the cells were differentiated to oligodendrocytes and neurons, with medium changes every 2–3 days for 14 days. At 21 days, cells were randomly divided into two groups. (A) In the OGD group, cells were cultured in a glucose-free medium and subjected to hypoxia (less than 1% O_2_, 5% CO_2_) for 3 h. Cells were then re-oxygenated (21% O_2_, 5% CO_2_). (B) In the control group, cells were cultured under normal oxygen conditions (21% O_2_, 5% CO_2_). In both groups, cells were assayed for HIF-1 (hypoxia-inducible factor) (1:300; BD Bioscience). At 22 days, cells subjected to OGD or normoxia were treated with PBS or with MSC-derived EVs (10 μg, 20 μg, 30 μg, 50 μg, 100 μg, or 200 μg). The cells were then fixed 25 days after seeding (Fig. 1b).

### In vitro co-labeling of EVs and neural stem cells

EVs were prelabeled with DiI (in red) and cells were prelabeled with DiO (in green). Timelabs were captured during 6 h after EV administration using the NIS-Element AR (Nikon) 4.5 Program.

### In vitro cell proliferation and immunofluorescence analyses

Cell proliferation was analyzed by Ki-67 staining (1:100, Chemicon, Temecula, CA, USA) using a × 40 objective lens and a Microscope Axio Lab.A1 (Carl Zeiss Microscopy GmbH, Germany).

The expression of various markers was studied using immunofluorescence for a marker related to mature neurons, such as microtubule-associated protein 2 (MAP-2) (1:1000, Millipore), and myelin-oligodendrocyte glycoprotein (MOG) (1:100, Abcam) for oligodendrocytes, followed by goat anti-mouse and anti-rabbit Alexa Fluor 488 and 594 (1:750, Invitrogen). Images were acquired as a maximum confocal projection and examined using a Leica TCS-SPE spectral confocal microscope (Leica Microsystems, Heidelberg, Germany) using a × 40 objective lens. The confocal images were obtained using LAS AF software (Leica). Mean fluorescence intensity was measured by the NIS-Element AR (Nikon) 4.5 Program.

### In vivo biodistribution study of EVs

Three animals per group were used for the evaluation of EV biodistribution after administration. The EVs were labeled with CD63 (1:250, Abcam) and DiI (Celltracker CM-DiI, Invitrogen). Cryosections (10 μm thick) of the brain, lung, liver, and kidney were counterstained with 4′,6-diamidino-2-phenylindole (DAPI), and biodistribution was analyzed by immunofluorescence staining at 48 h post-stroke. Images were acquired as a confocal maximum projection using a Leica TCS-SPE confocal microscope (Leica Microsystems, Heidelberg, Germany), with a × 40 objective lens, and analyzed using LAS AF software (Leica). Mean fluorescence intensity was measured by the NIS-Element AR (Nikon) 4.5 Program (Fig. 1c).

### Animals and surgery

Adult male and female rats were subjected to a subcortical infarction by an injection of 1 μL endothelin-1 (Calbiochem) (0.25 μg/μl) into the striatum using the following coordinates: + 0.4 mm anteroposterior, + 3.5 mm lateral, and + 6 mm dorsoventral from the bregma. Full details of this model have previously been published [13].

A total of 55 male and female Sprague-Dawley rats (8–9 weeks old, weighing 200–250 g) were used for the entire study. Our strategy for animal welfare includes Reduction of Animals in Research. According to the 3Rs strategy [22] and based on the results of the in vitro study (positive results for at least 50 μg of EVs henceforth), we decided to conduct the in vivo study of the following study groups (*n* = 10 [5 male and 5 female]) in each group: the sham rats were subjected to surgery without subcortical infarction and 1 ml of intravenous saline administration; the control rats were subjected to a subcortical infarction and 1 ml of intravenous saline administration; and three groups of rats were subjected to a subcortical infarction with various doses of intravenous EVs administration called low dose, 50 μg; intermediate dose, 100 μg; and high dose, 200 μg. EV treatment was administered via the tail vein at 24 h after surgery. All the animals were euthanized 28 days post-stroke (Fig. 1d). Five rats were excluded from the study: one male rat died during the magnetic resonance imaging (MRI), another male rat died 7 days after infarction surgery (from the control group), and the other three rats (one male and two females) were excluded because they did not show lesions on MRI analysis. Additionally, three animals per group were used for biodistribution analysis, which were euthanized at 48 h after stroke.

### Quantification of circulating EVs in serum

In order to analyze whether EV administration modifies the circulating number of EVs, this EV has been analyzed 24 h before stroke induction and then 48 h and 28 days after surgery. For this analysis, the blood samples were drawn from the tail vein and collected in anti-coagulant tubes, which were then centrifuged at 3000 rpm for 15 min. Serum samples were collected and immediately stored at − 80 °C until analysis. EVs were isolated from serum samples using an EV extraction kit (exoQuick, SBI). A commercially available enzyme-linked immune sorbent assay kit (CD63-A, SBI System Bioscience) was used by a researcher who was blinded to the study group to which the samples belonged, to evaluate differences in the quantity of circulating EVs in serum between groups.

### Functional evaluation scales

Functional evaluations were performed for all the animals by a blinded observer at 48 h, 7 days and 28 days after stroke induction. Motor performance was evaluated using the rotarod, beam-walking, and Rogers tests. The rotarod test measured the time to fall from a rotating cylinder [23]. The beam-walking test measured the rats’ ability to walk along a wooden beam (2.5 × 2.5 × 80 cm). Scores were assigned as follows: 0, traversed the beam with no foot slip; 1, traversed with grasping the side of the beam; 2, difficulty crawling along the beam but able to traverse; 3, required > 10 s to traverse the beam because of difficulty walking; 4, unable to traverse the beam; 5, unable to move the body or any of its limbs along the beam; and 6, unable to stay on the beam for > 10 s [24]. A variant of Rogers’ functional scale was used to assign scores as follows: 0, no functional deficit; 1, failure to fully extend forepaw; 2, decreased grip of forelimb while tail gently pulled; 3, spontaneous movement in all directions, contralateral circling only if pulled by the tail; 4, circling; 5, walking only when stimulated; 6, unresponsive to stimulation with a depressed level of consciousness; and 7, dead [25].

### In vivo magnetic resonance imaging

Lesion size was determined by MRI at 48 h and 28 days after subcortical infarction using a 7-T horizontal bore magnet (Bruker Pharmascan, Ettlingen, Germany) equipped with a 1H circular polarized volume coil with inner diameter of 40 mm and a Bruker gradient insert with 90 mm of diameter (maximum intensity 30 G/cm) and a T2-weight spin-echo image. Animals were anesthetized with a 2% isoflurante-oxygen mixture in an induction chamber, and the flow of anesthetic gas was constantly regulated to maintain a breathing rate of 50 +/− 20 bpm. The animal temperature was maintained at approx. 37 °C with a hot water circulated blanket. The physiological state of the rats was monitored using a monitoring system by SA INstruments (Stony Brook, NY; http://www.i4sa.com/) that controlled the respiratory rate and body temperature.

The lesion area was expressed as a percentage of the contralateral hemisphere, after correcting for brain edema. To correct for the brain edema effect, the lesion volume was determined by an indirect method: (infarct area) = (area of the intact contralateral hemisphere) − (area of the intact ipsilateral hemisphere). For the tractography analysis, diffusion tensor data were acquired with a spin-echo single-shot echo-planar imaging (EPI) pulse sequence using the following parameters: TR/TE 8000/35 ms; a signal average of 12, 30 noncollinear diffusion gradient scheme with a diffusion weighting *b* = 500 s/mm2 and *b* = 1000 s/mm2, 18 slices with a slice thicknes of 1.5 mm without a gap, field of view 35 × 35 mm. Total imaging time was 1 h 44 min. All EPI data were acquired with a single-shot EPI sequence, 96 × 96 matrix, and zero filled in *k* space to construct a 128 × 128 image matrix. Fractional anisotropy, mean diffusivity, trace, and the eigenvalues and eigenvector maps were calculated with a homemade software application written in Matlab (R2007a), and the 3D fiber tract map was created using the MedINRIA DTI Track software (https://med.inria.fr/the-app/downloads) [24]. DTI was performed 48 h and at 28 days after treatment. The researchers recording the data were entirely blinded to the experimental groups.

### Cell death and proliferation

At 28 days, cell death was determined in the peri-infarct zone using TUNEL staining (TdT-FragEL DNA Fragmentation Detection Kit, Oncogene Research Products). Cell counts were performed in 4 sections from each animal (*n* = 6 per group; 3 males and 3 females) based on their nuclear morphology and dark color using a × 40 objective lens and image analysis software (Microscope Axio Lab.A1, Carl Zeiss Microscopy GmbH, Germany) as previously described [7].

Cell proliferation was analyzed using Ki-67 staining (1:100, Chemicon, Temecula, CA, USA) in the subventricular zone after 28 days, in 4 sections of each animal (*n* = 6 per group; 3 males and 3 females), selected as previously described [7], using a × 40 objective lens and a Microscope Axio Lab.A1 (Carl Zeiss Microscopy GmbH, Germany).

The differentiation of proliferating cells was analyzed using co-staining with Ki-67 (1:400, Millipore) followed by goat anti-rabbit Alexa Fluor 488 antibody (1:750, Invitrogen) and doublecortin (DCX) (1:250, Santa Cruz), Olig-2 (1:400, Millipore), and glial fibrillary acidic protein (GFAP) (1:400, Chemicon), followed by goat anti-mouse Alexa Fluor 594 antibody (1:750, Invitrogen). Images were acquired as a confocal maximum projection using a Leica TCS-SPE confocal microscope (Leica Microsystems, Heidelberg, Germany), with a × 40 objective lens, and analyzed using LAS AF software (Leica). Mean fluorescence intensity was measured by the NIS-Element AR (Nikon) 4.5 Program.

### Immunofluorescence analyses

The lesion area was evaluated using immunofluorescence for oligodendrocytes with MOG (1:100, Abcam) and myelin basic protein (MBP) (1:100, Abcam); astrocytes with GFAP (1:400, Chemicon); synaptic plasticity with synaptophysin (SYP) (1:200, Sigma); and trophic factor with brain-derived neurotrophic factor (BDNF) (1:1000, Millipore), followed by goat anti-mouse and anti-rabbit Alexa Fluor 488 (1:750, Invitrogen). Images were acquired as a confocal maximum projection using a Leica TCS-SPE confocal microscope (Leica Microsystems, Heidelberg, Germany), with a × 40 objective lens, and analyzed using LAS AF software (Leica). To quantify the expression of brain repair-associated markers, the mean fluorescence intensity was obtained using the NIS-Element AR (Nikon) 4.5 Program. The experiments, images, and quantification of the samples were performed by blinded observers using the same microscope configurations to eliminate bias due to background normalization (4 animals for each group [2 males and 2 females]; 4 sections in each animal per group).

### Statistical analysis

The results were expressed as mean ± standard deviation (SD) and the data were tested for normality using the Shapiro-Wilk test. Normal distributed data was analyzed using one-way ANOVA followed by the Tukey HSD test (in vitro study, EVs in serum, Ki67, GFAP). Non-normal distributed data was analyzed using Kruskal-Wallis test followed by the Mann-Whitney test (MRI T2 and tractography, FE, EVs biodistribution, TUNEL, MOG, MBP, SYP, BDNF). Values of *p* < 0.05 were considered significant at a 95% confidence interval; the data were analyzed using the statistical IBM SPSS 23 program and GraphPad Prism 8 software. The power analysis showed that with non-parametric testing, at least 10 animals needed to be randomly assigned to each group to achieve a significance level of 5% (alpha) and a power of 80% (1-beta).

## Results

### EV characterization

EVs showed typical morphology and size (< 200 nm) by an electron microscope and Nanosight. EVs were detected by specific positive (CD63, CD81, ALIX) and negative (albumin) markers on a western blot assay (Fig. 1a).

### In vitro analysis under conditions of normoxia and OGD

The expression of HIF-1 in cultured cells confirmed that they were subjected to OGD properly (Supplemental Material).

### EVs and neural stem cell co-labeling

**Fig. 2:**
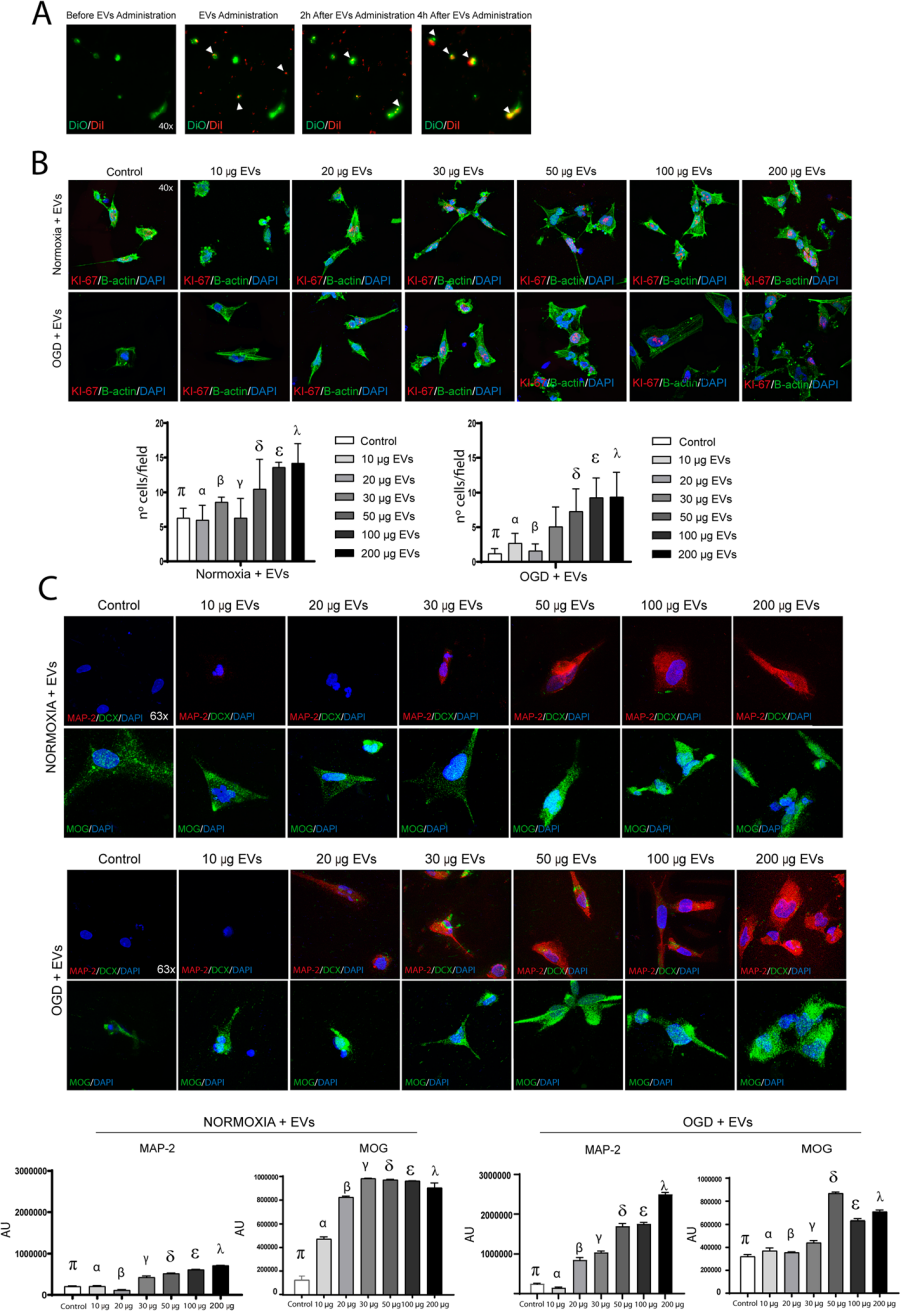
In vitro assay. **a** Timelabs images of biodistribution of the EVs-DiI in neural stem cells-DiO subjected to OGD before, in the moment of EVs administration 2 h and 4 h after EVs administration. **b** Qualitative and quantitative images of cell proliferation of neural stem cells by Ki-67 staining under conditions of normoxia and OGD (*n* = 10 assays per group) (data are mean ± SD; for normoxia conditions *p* < 0.05: *π* = control vs. 100 μg and 200 μg; *α* = 10 μg, vs. 100 μg and 200 μg; *β* = 20 μg, vs. 100 μg and 200 μg; *γ* = 30 μg vs. 100 μg and 200 μg; *δ* = 50 μg vs. 10 μg and 30 μg; *ε* = 100 μg vs. control, 10 μg, 20 μg, and 30 μg; *λ* = 200 μg vs. control, 10 μg, 20 μg, and 30 μg. For OGD conditions, *p* < 0.05: *π* = control vs. 50 μg, 100 μg, and 200 μg; *α* = 10 μg, vs. 100 μg and 200 μg; *β* = 20 μg, vs 50 μg, 100 μg, and 200 μg; *δ* = 50 vs. control and 20 μg; ε = 100 vs. control, 10 μg and 20 μg; *λ* = 200 vs. control, 10 μg and 20 μg; 4′,6-diamidino-2-phenylindole (DAPI) is used for nuclear staining and B-actin for cytoplasm staining. Abbreviations: EVs, extracellular vesicles; OGD, oxygen and glucose deprivation. **c** Representative immunofluorescence images of neurons expressing MAP-2 and oligodendrocyte expressing MOG after receiving various doses of EVs under conditions of normoxia and OGD. 4′,6-diamidino-2-phenylindole (DAPI) was used for nuclear staining. Quantitative analysis of MAP-2 and MOG marker expression by immunofluorescence (*n* = 10 assays per group) (data are mean ± SD. For MAP-2 marker in normoxia conditions *p* < 0.05: *π* = control vs. 20 μg, 30 μg, 50 μg, 100 μg, and 200 μg; *α* = 10 μg, vs. 20 μg, 30 μg, 50 μg, 100 μg, and 200 μg; *β* = 20 μg, vs. all of groups; *γ* = 30 μg vs. all of groups; *δ* = 50 μg vs. all of groups; *ε* = 100 μg vs. all of groups; *λ* = 200 μg vs. all of groups. For MOG marker in normoxia conditions, *p* < 0.05: *π* = control vs. all of groups; *α* = 10 μg, vs. all of groups; *β* = 20 μg, vs. all of groups; *γ* = 30 μg vs. control, 10 μg; 20 μg; *δ* = 50 μg vs. control, 10 μg; 20 μg; *ε* = 100 μg vs. control, 10 μg; 20 μg; *λ* = 200 μg vs. control, 10 μg; 20 μg. For MAP-2 marker in OGD conditions, *p* < 0.05: *π* = control vs 50 μg, 100 μg, and 200 μg; *α* = 10 μg, vs. 20 μg, 30 μg, 50 μg, 100 μg, and 200 μg; *β* = 20 μg, vs 10 μg; 30 μg; 50 μg, 100 μg, and 200 μg; *γ* = 30 μg vs. 10 μg; 20 μg; 50 μg, 100 μg, and 200 μg; *δ* = 50 μg vs. control, 10 μg; 20 μg; 30 μg and 200 μg; *ε* = 100 μg vs. control, 10 μg; 20 μg, 30 μg, and 200 μg; *λ* = 200 μg vs. all of groups. For MOG marker in OGD conditions, *p* < 0.05: *π* = control vs. 30 μg, 50 μg, 100 μg, and 200 μg; *α* = 10 μg, vs. 30 μg, 50 μg, 100 μg, and 200 μg; *β* = 20 μg, vs. 30 μg, 50 μg, 100 μg, and 200 μg; *γ* = 30 μg vs. all of groups; *δ* = 50 μg vs. all of groups; *ε* = 100 μg vs. all of groups; *λ* = 200 μg vs. all of groups). Abbreviations: DCX, doublecortin; EVs, extracellular vesicles; MAP-2, microtubule associated protein 2; MOG, myelin oligodendrocyte glycoprotein; OGD, oxygen and glucose deprivation

For biodistribution analysis, EVs were prelabeled with DiI tracker (EVs-DiI) and neural stem cells were prelabeled with DiO (cells-DiO). EVs-DiI were administered in vitro to cells-DiO subjected to OGD. There was no co-localization observed between EVs-DiI and cells-DiO 2 h after administration; however, there was co-labeling of DiI and DiO markers 4 h after EVs administration (Fig. 2a).

### Effect of EV treatment on cell proliferation

A quantitative analysis of proliferative cells was performed using Ki-67 labeling. Under conditions of normoxia, the number of Ki-67-positive cells was significantly higher in the cell receiving 100 μg (12.1 ± 0.70 cells; *p* = 0.001) and 200 μg (14.2 ± 2.82 cells *p* < 0.001) compared to the control cells (6.3 ± 1.41 cells). However, no significant differences were found in the cells receiving 10 μg (6.0 ± 2.12 cells), 20 μg (8.6 ± 0.70 cells), 30 μg of EVs (6.3 ± 2.82 cells), and 50 μg (10.5 ± 4.24 cells) compared to the controls (6.3 ± 1.41 cells). Under OGD conditions, cell proliferation was significantly higher in the cells receiving 50 μg of EVs (7.3 ± 3.25 cells; *p* = 0.005), 100 μg (9.3 ± 2.82 cells; *p* < 0.001), and 200 μg (9.4 ± 3.53 cells; *p* < 0.001) compared to control cells (1.2 ± 0.7 cells). However, no significant differences were found in cell proliferation when comparing 10 μg (2.7 ± 1.41 cells), 20 μg (1.6 ± 0.98 cells), and 30 μg doses of EVs (5.1 ± 2.82 cells) with the control cells (1.2 ± 0.7 cells) (Fig. 2b).

### Effect of EV treatment on brain repair-associated marker expression

The expression of the MAP-2 marker was similar in control cells [2.09 × 10^5^ ± 1.15 × 10^4^ arbitrary units (A.U.)] than in cells receiving 10 μg of EVs (2.12 × 10^5^ ± 1.5 × 10^4^ A.U.). MAP-2 signal was significantly lower in cells treated with 20 μg of EVs (1.16 × 10^5^ ± 1.4 × 10^4^ A.U.; *p* = 0.011) compared to control cells (2.09 × 10^5^ 1.15 × 10^4^ A.U.). MAP-2 signal was significantly higher in cells receiving 30 μg (4.28 × 10^5^ ± 2.9 × 10^4^ A.U.; *p* < 0.001), 50 μg (5.21 × 10^5^ ± 1.07 × 10^4^ A.U.; *p* < 0.001), 100 μg (6.14 × 10^5^ ± 6.9 × 10^4^ A.U.; *p* < 0.001), and 200 μg doses of EVs (7.14 × 10^5^ ± 4.9 × 10^4^ A.U.; *p* < 0.001) compared to controls (2.09 × 10^5^ 1.15 × 10^4^ A.U.) under conditions of normoxia (Fig. 2c).

Notably, under OGD conditions, the expression of MAP-2 was similar in control cells (2.40 × 10^5^ ± 2.06 × 10^4^ A.U.), in cells receiving 10 μg (1.40 × 10^5^ ± 2.5 × 10^4^ A.U.), 20 μg (8.4 × 10^5^ ± 7.2 × 10^4^ A.U.), and 30 μg doses of EVs (10.3 × 10^5^ ± 4.2 × 10^4^ A.U. However, a significant increase in the signal of MAP-2 marker was found in cells treated with 50 μg (16.99 × 10^5^ ± 7.4 × 10^4^ A.U.; *p* < 0.001), 100 μg (17.57 × 10^5^ ± 4.4 × 10^4^ A.U.; *p* < 0.001), and 200 μg doses of EVs (24.95 × 10^5^ ± 5.62 × 10^4^ A.U.; *p* < 0.001) (Fig. 2c).

MOG expression in control cells (1.37 × 10^5^ ± 1.30 × 10^4^ A.U.; *p* < 0.001) was significantly lower than all the groups of cells receiving all doses of EVs. However, the signal of MOG marker was significantly higher in cells treated with 30 μg (9.8 × 10^5^ ± 0.2 × 10^4^ A.U.), 50 μg (9.69 × 10^5^ ± 0.4 × 10^4^ A.U.), 100 μg (9.60 × 10^5^ ± 0.1 × 10^4^ A.U.), and 200 μg doses of EVs (9.02 × 10^5^ ± 4.08 × 10^4^ A.U.) when comparing to the cells receiving 10 μg (4.72 × 10^5^ ± 1.76 × 10^4^ A.U.; *p* < 0.001) and 20 μg of EVs (8.22 × 10^5^ ± 0.8 × 10^4^ A.U.; *p* = 0.006) under conditions of normoxia (Fig. 2c).

Under OGD conditions, the signal of MOG marker was similar in control cells (3.20 × 10^5^ ± 1.73 × 10^4^ A.U.) than in 10 μg (3.70 × 10^5^ ± 2.4 × 10^4^ A.U.) and 20 μg of EVs (3.55 × 10^5^ ± 0.5 × 10^4^ A.U.). However, the signal of MOG marker was significantly higher in cells treated with 30 μg (4.40 × 10^5^ ± 1.8 × 10^4^ A.U.; *p* = 0.012), 50 μg (8.69 × 10^5^ ± 0.9 × 10^4^ A.U.; *p* < 0.001), 100 μg (6.33 × 10^5^ ± 1.4 × 10^4^ A.U.; *p* < 0.001), and 200 μg of EVs (7.09 × 10^5^ ± 1.3 × 10^4^ A.U.; *p* < 0.001) compared to controls (Fig. 2c).

Taking into account the results of the in vitro study, it was concluded that doses of 10 μg, 20 μg, and 30 μg of EVs are not enough to promote proliferation and differentiation in OGD conditions of different lineages of brain cells. In order to minimize the number of animals used in research, we eliminated those doses from the in vivo study.

## Analysis of EV treatment in an in vivo animal model of stroke

### EV biodistribution in tissue and serum

**Fig. 3:**
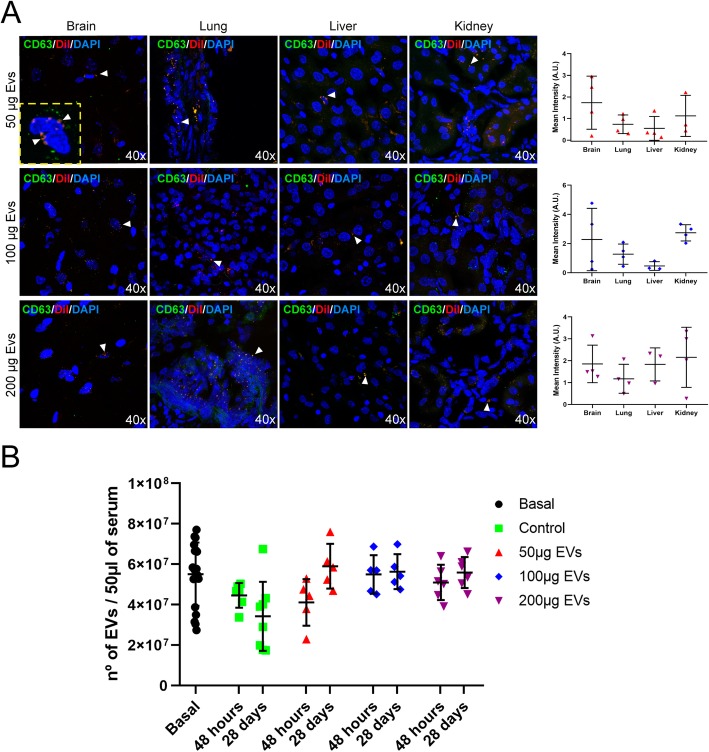
EV biodistribution and quantification. **a** Representative immunofluorescence images displaying the biodistribution of intravenously administered EVs in the various organs: brain, lung, liver, and kidney (red color shows DiI marker EVs, green color shows CD63 marker) and their quantification (data are shown as mean ± SD; *n* = 3 animals per group). **b** Quantification of circulating EVs in serum in the groups treated with one of several doses of EVs before stroke (baseline) and at 48 h and 28 days after stroke (*n* = 10 animals per group; 5 male and 5 female). Data are shown as mean ± SD

Following the analysis of histochemical sections of various organs, the EVs-DiI were found not only in peripheral organs such as the lung, liver, and kidney, but also in the brain tissue 48 h after stroke (Fig. 3a). Quantification of EVs in serum by ELISA analysis showed a tendency of EVs to decrease after 28 days (3.42 × 10^7^ ± 1.70 × 10^7^) compared to the levels after 48 h (4.45 × 10^7^ ± 0.61 × 10^7^) in the control animals. The number of EVs in the serum of the animals receiving treatment with EVs did not show significant differences by 28 days (low dose, 5.83 × 10^7^ ± 1.26 × 10^7^; intermediate doses, 5.57 × 10^7^ ± 0.98 × 10^7^; high dose, 5.56 × 10^7^ ± 1.49 × 10^7^) compared to the levels after 48 h (low dose, 4.19 × 10^7^ ± 1.31 × 10^7^; intermediate dose, 5.44 × 10^7^ ± 1.09 × 10^7^; high dose, 5.24 × 10^7 ^± 1.05 × 10^7^) after treatment (Fig. 3b).

### Effect of EV treatment on functional recovery

**Fig. 4:**
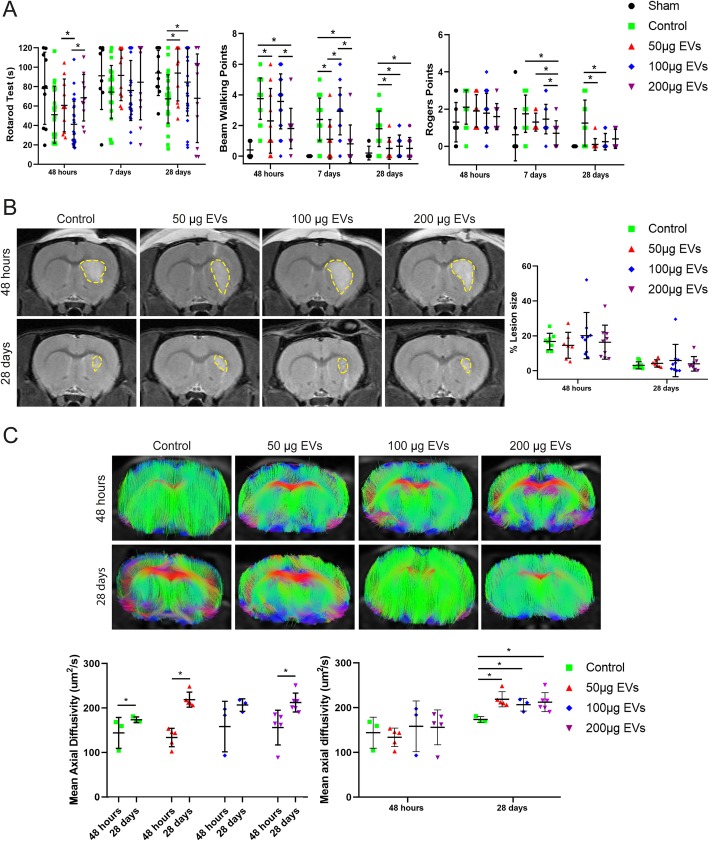
Functional recovery and MRI analysis. **a** Recovery of motor impairment analysis of functional evaluation using three different tests (rotarod, beam-walking, and Rogers test) in animals subjected to three doses of EV treatment (*n* = 10 animals per group; 5 males and 5 females). **b** Representative images of T2-weighted MRI of animals receiving all doses of EVs (*n* = 6 animals per group: 3 males and 3 females). **c** Representative tractography images with the lesion side in detail of the animals subjected to various doses of EVs (*n* = 6 animals per group; 3 males and 3 females). Data are shown as mean ± SD. *p < 0.05. Abbreviations: EVs, extracellular vesicles

The functional evaluation of the sham animals was homogeneous throughout the study. At 28 days, the animals treated with EVs showed a significant improvement in functional evaluation compared to the control group using a low dose of EVs (rotarod [control, 67.43 ± 24.43 s; low dose, 94.04 ± 28.80 s; *p* = 0.026], beam-walking [control, 1.79 ± 1.17 points; low dose, 0.50 ± 0.71 points; *p* = 0.003], and Rogers [control, 1.25 ± 1.24 points; low dose, 0.10 ± 0.32 points; *p* = 0.006]); using an intermediate dose of EVs (rotarod [control, 67.43 ± 24.43 s; intermediate dose, 84.76 ± 34.84 s; *p* = 0.046], beam-walking [control, 1.79 ± 1.17 points; intermediate dose, 0.64 ± 0.73 points; *p* = 0.001], and Rogers [control, 1.25 ± 1.24 points; intermediate dose, 0.25 ± 0.44 points; *p* = 0.001]); and using a high dose of EVs (beam walking [control, 1.79 ± 1.17 points; high dose, 0.50 ± 0.71 points; *p* = 0.003]. But there was no significant difference found in the rotarod test at 28 days between the control group (67.43 ± 24.43 s) and the high-dose EV group (68.07 ± 45.65 s). Despite the fact that all the treated groups achieved a significant recovery after 28 days compared to the control group, the animals that received a high dose, in some cases, showed the earliest improvement in recovery at 48 h and 7 days (data not show). However, after 28 days, the high-dose group did not show significant differences in the rotarod, beam-walking, and Rogers tests compared to intermediate and low doses (Fig. 4a).

### Effect of EV treatment on lesion size and tract connectivity

The lesion size viewed on MRI in the EV-treated animals was indistinguishable from the size of the lesions in the control group (3.04% ± 2.01%) compared with the treated groups at 28 days (low dose, 4.15% ± 2.06%; intermediate dose, 5.83% ± 9.26%; high dose, 3.90% ± 4.16%) (Fig. 4b). On DTI tractography, significant higher axial diffusivity values were found in control (173.68 ± 6.42 μm^2^/s vs. 144.06 ± 34.68 μm^2^/s; *p* = 0.05), low (218.87 ± 17.03 μm^2^/s vs. 133.76 ± 20.73 μm^2^/s; *p* = 0.009), and high (204.77 ± 10.94 μm^2^/s vs. 156.21 ± 38.98 μm^2^/s; *p* = 0.0006) doses of EVs when comparing at 28 days with 48 h, respectively. Besides, at 28 days, significant differences were observed between the control animals (173.68 ± 6.42 μm^2^/s) and the animals receiving treatment with various doses of EVs (low dose, 218.87 ± 17.03 μm^2^/s, *p* = 0.025; intermediate dose, 206.80 ± 14.11 μm^2^/s, *p* = 0.050; high dose, 204.77 ± 10.94 μm^2^/s, *p* = 0.020) in axial diffusivity (Fig. 4C). In the treated groups, a significant progressive reduction in white matter injury showed augmented connectivity of fiber tracts compared to the control group.

### Effect of EV treatment on cell death and proliferation

**Fig. 5:**
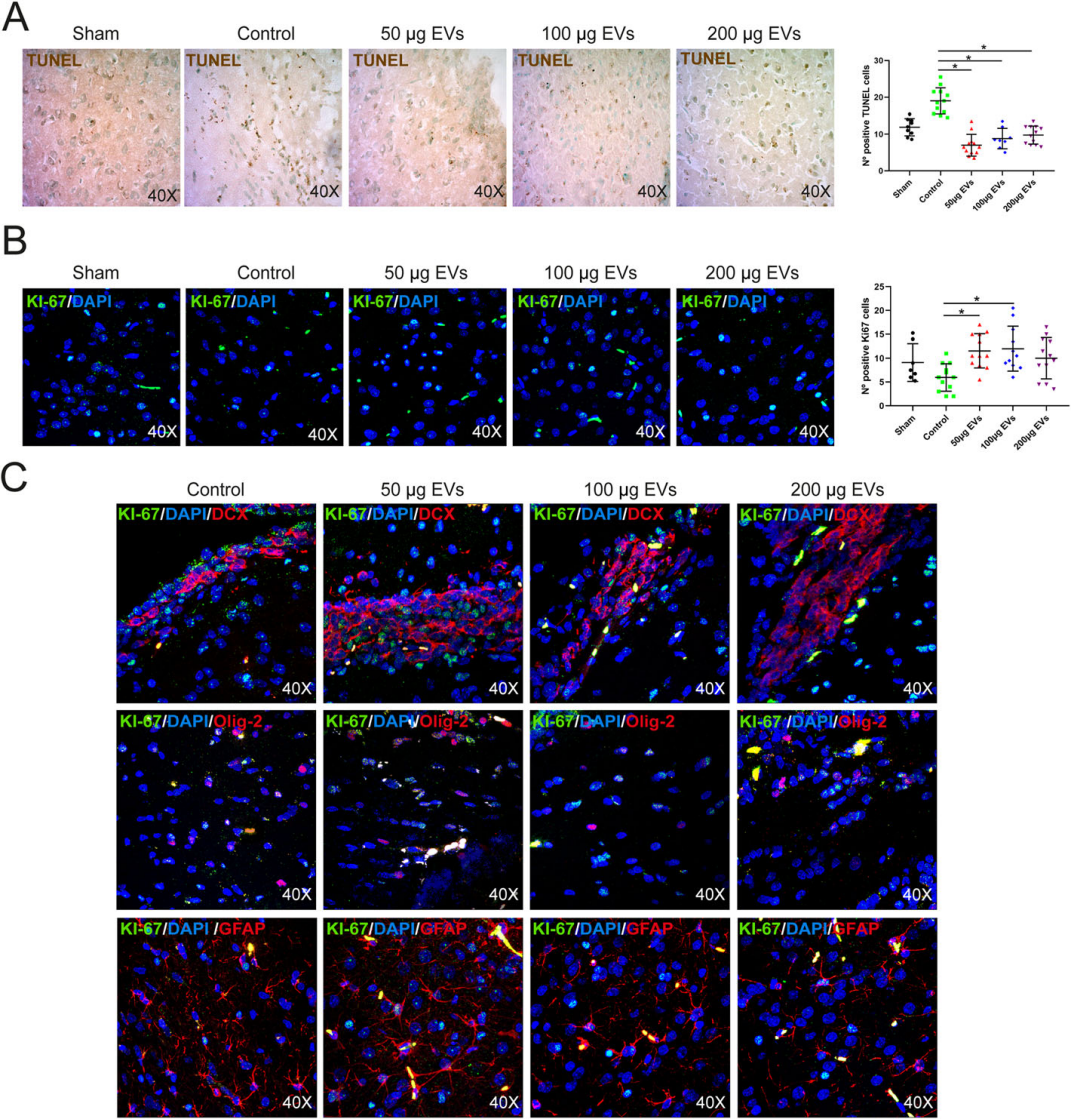
Cell death and proliferation analysis. **a** Qualitative and quantitative images of cell death by TUNEL [6 animals: 3 males and 3 females], 4 sections each per group. Data are mean ± SD; *p < 0.05. **b** Qualitative and quantitative images of cell proliferation by Ki-67 staining [6 animals: 3 males and 3 females], 4 sections each per group. Data are mean ± SD; *p < 0.05. **c** Representative images of proliferating cells co-labeled with DCX, Olig-2, and GFAP markers at 28 days after treatment; DAPI was used for nuclear staining. Abbreviations: DAPI, 4′,6-diamidino-2-phenylindole; EVs, extracellular vesicles

After 28 days, there were significantly fewer TUNEL-positive cells in the peri-infarct area in the treatment groups (low dose, 7.00 ± 2.95 cells, *p* = 0.001; intermediate dose, 8.81 ± 2.79 cells, *p* = 0.001; high dose, 9.75 ± 2.46 cells, *p* = 0.001) than in the control group (19.00 ± 3.55 cells) (Fig. 5a).

A quantitative analysis of proliferative cells was performed using Ki-67 labeling 28 days after treatment. The number of Ki-67-positive cells was significantly higher in the treatment groups (low dose, 11.54 ± 3.58 cells, *p* = 0.004; intermediate dose, 12.00 ± 4.71 cells, *p* = 0.005) than in the control group (5.96 ± 2.90 cells) (Fig. 5b).

In all groups, especially in treated groups with EVs, proliferating cells with various markers were found to be co-labeled, such as with DCX, Olig-2, and GFAP, indicating a new generation of several types of neurovascular unit cells (Fig. 5c).

### Effect of EV treatment on the expression of brain repair-associated marker

**Fig. 6:**
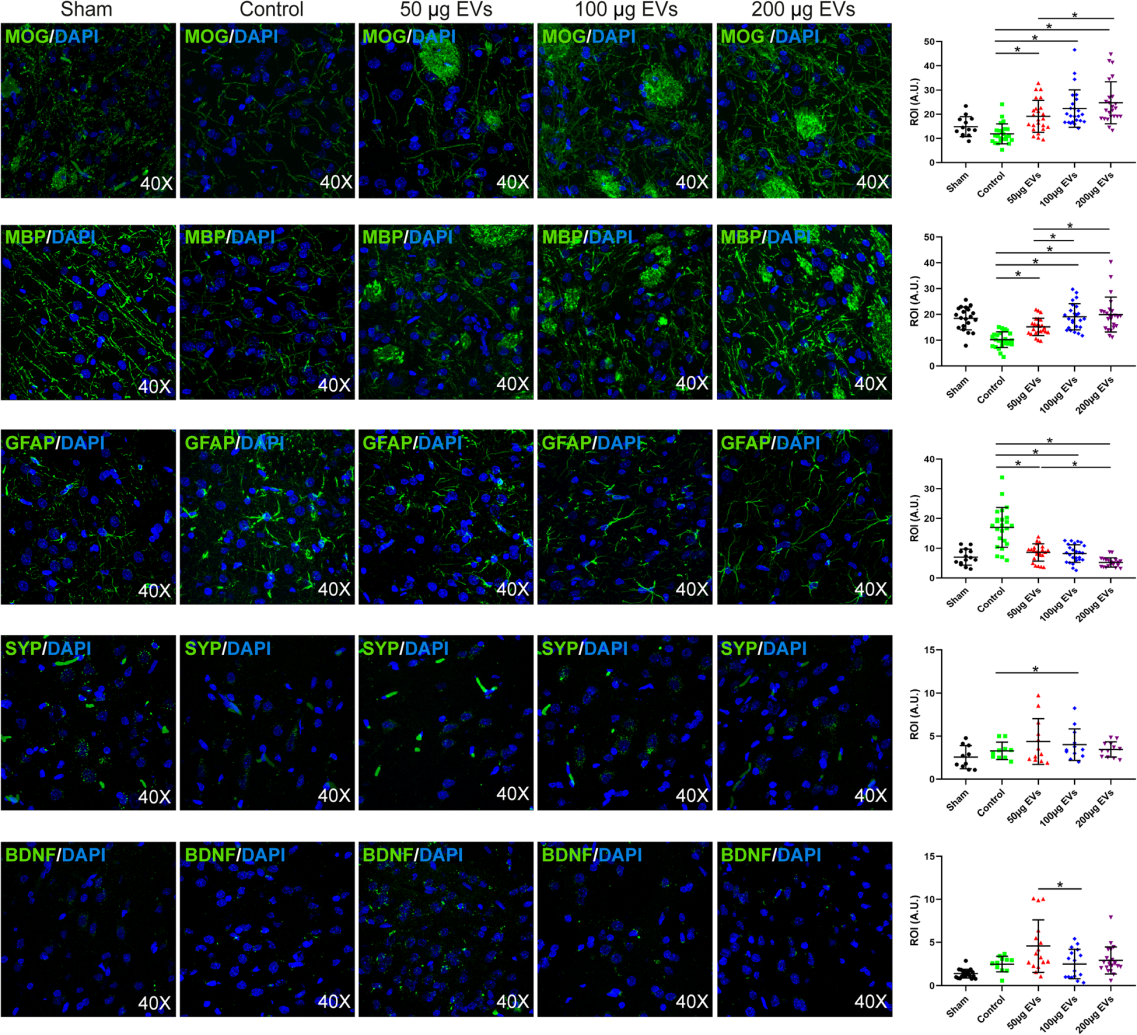
Brain repair-associated markers. Representative immunofluorescence images of MOG, MBP, GFAP, SYP, and BDNF (4 animals for each group [2 males and 2 females], 4 sections in each animal per group). Data are mean ± SD, *p < 0.05. Abbreviations: EVs, extracellular vesicles

Considering the heterogeneity of EV contents, brain repair enhancement is expected to be mediated by various mechanisms. For this reason, this study analyzed the expression of several brain repair-associated markers related to all components of the neurovascular unit (neurons, astrocytes, oligodendrocytes, myelin, vessels, etc.) (Fig. 6).

Regarding white matter-related markers, levels of MOG were significantly higher in the treatment groups (low dose, 19.15 ± 6.64 A.U., *p* = 0.001; intermediate dose, 22.38 ± 7.76 A.U., *p* = 0.001; high dose, 24.77 ± 8.70 A.U., *p* = 0.001) than in the control group (11.88 ± 4.13 A.U.). The animals treated with EVs showed significantly higher levels of MBP (low dose, 15.18 ± 3.36 A.U., *p* = 0.001; intermediate dose, 19.10 ± 5.08 A.U., *p* = 0.001; high dose, 19.95 ± 6.77 A.U., *p* = 0.001) than those in the control group (10.23 ± 3.04 A.U.) With respect to astrocytes, GFAP levels were significantly lower in the treatment groups (low dose, 8.65 ± 2.92 A.U., *p* = 0.001; intermediate dose, 8.24 ± 2.97 A.U., *p* = 0.001; high dose, 5.21 ± 1.49 A.U., *p* = 0.001) than in the control group (17.04 ± 6.66 A.U.). Neurons showed significantly higher synaptophysin levels in the treatment group with an intermediate dose (3.63 ± 1.26 A.U., *p* = 0.047) than in the control group (2.45 ± 0.23 A.U.).

Conversely, with respect to the trophic factor BDNF, only in the low-dose group (5.05 ± 2.96 A.U.) were its levels significantly higher than in the control (2.50 ± 0.88 A.U., *p* = 0.031) and the intermediate groups (2.50 ± 1.69 A.U., *p* = 0.046). BDNF signal was similar in the intermediate (2.50 ± 1.69 A.U.) and high dose (2.91 ± 1.56 A.U.) groups compared to the control group (2.50 ± 0.88 A.U.).

## Discussion

Treatment with MSC-derived EVs is an emerging, powerful and promising therapy to mediate restorative effects in neurological disease, including stroke. The treatment appears to act by carrying multiple active molecules that participate in enhancing brain repair after stroke [8, 9]. However, many aspects of this treatment, such as the most suitable dose, remain unknown and need to be determined before the treatment can be routinely applied in clinical practice. Therefore, the aim of this study was to identify the optimal effective dose using different doses of EVs (10 μg, 20 μg, 30 μg, 50 μg, 100 μg, and 200 μg) that could promote proliferation and differentiation in an in vitro model of OGD conditions in neurons and oligodendrocytes and could enhance brain protection, repair, and recovery in an experimental animal model of subcortical infarction. The in vitro bioactivity study showed that at least a dose of 50 μg of EVs was needed to increase cell proliferation under OGD conditions in neural stem cells. In this regard, at least the same dose of EVs (50 μg) is needed to induce a greater number of differentiation markers to neurons (MAP-2) and oligodendrocytes (MOG) under OGD conditions. Taking into account the results of the in vitro study, the authors concluded that doses 10 μg, 20 μg, and 30 μg of EVs were not enough to promote proliferation and differentiation under OGD conditions of different lineages of brain cells. In order to minimize the number of animals used in research, those doses were excluded from the in vivo study. Thus, after an in vivo analysis of an intravenous infusion of 50 μg, 100 μg, and 200 μg of EVs, all these doses were shown to improve functional outcomes associated with decreased cell death, as well as restoration of fiber tract connectivity, increasing oligodendrocyte markers, and re-myelination in an experimental animal model of subcortical infarction.

One of the characteristics that make EVs an attractive treatment for stroke is their ability to cross the BBB following intravenous administration. In order to amplify the information regarding biodistribution, pre-labeled intravenously administered EVs were tracked in the brain, peripheral organs, and serum of rats. In this study, pre-labeled EVs were found in the brain tissue and peripheral organs (lung, liver, and kidney) 48 h after stroke. These results are consistent with previous studies demonstrating that intravenously administered EVs can reach not only peripheral organs but also the brain, co-labeling with VEGF, NeuN, GFAP, and Iba-1 [13], and that the intravenously administered GFAP-tagged EVs were transferred to adjacent astrocytes and neurons [26]. These results were complemented by those obtained in the in vitro study, which showed that EVs are internalized in the cells 4 h after their administration.

Taking into account the translational point of view, when a new stroke strategy is tested on animals, it is important to know whether it has an effect on functional recovery. In this study, the animals receiving high doses of EVs achieved the earliest improvement compared to those receiving low and intermediate doses. In contrast, only low and intermediate doses achieved good functional recovery at 28 days compared to the controls. At 28 days, these results clearly suggest a true upturn-enhancing effect for MSC-derived EV administration. These results are consistent with previous experimental data in which MSC-derived EVs administered alone or in combination with MSCs improved long-term neurological functional outcome after stroke [12–16].

Despite enhancing motor recovery, intravenously administered MSC-derived EVs did not reduce lesion volume in study as measured by T2-MRI. This result agrees with a previous finding in which the lesion size in the EV-treated animals was indistinguishable from the control [13]. In an MRI tractography analysis, we found a time-dependent increasement in DTI parameters after stroke in the peri-lesioned area in the control group, low and high doses of EVs groups. These results agree with previous studies which have demonstrated that sprouting of fibers from surviving neurons and formation of new synapses were found in peri-infarct zones after stroke a time-dependent manner [27, 28]. It is expected that the rats show some recovery, since axonal projections are reorganized in the perilesional tissue after stroke [29]. Moreover, DTI analysis also showed significantly higher levels of axial diffusivity inside the lesion area of those animals that had been treated with EVs compared to the controls. These results may indicate that although the overall lesion size did not decrease, inside the lesion, there was higher axonal connectivity and better recovery of white matter. Thus, EV treatment produces a significant enhancement of axonal repair and brain connectivity. The results of this study are consistent with those of previous studies which found an increase in axonal density in the cortical area [13] and neurite remodeling in the ischemic boundary [14].

Furthermore, this study also investigated the ability of various doses of MSC-derived EVs to reduce cell death after a subcortical infarction. In this regard, animals receiving any of the doses of EVs presented significantly fewer TUNEL-positive cells in the peri-infarct area after 28 days, compared to controls indicating that 50 μg of EVs is enough to reduce cell death under ischemic conditions.

The central nervous system continuously generates new cells in several specific regions of the adult mammalian brain, and in this study, said proliferation was shown to be enhanced by EVs derived from MSCs. Ki-67 staining showed large numbers of proliferative cells, using low and intermediate doses of EVs compared to the control group at 28 days after subcortical infarction. Consistent with previous studies [12, 14, 15], these results suggest that in the treated groups, proliferating cells with various markers were found to be co-labeled, for example, with DCX, GFAP, and Olig-2, indicating genesis of new neurons and astrocytes as well as oligodendrocyte progenitors. These results are also correlated with those obtained in vitro, where at least 50 μg of EVs were needed to promote proliferation of neural stem cells subjected to OGD conditions.

Some brain repair mechanisms are quickly activated after ischemia, and MSC-derived EVs offer a powerful therapeutic tool to promote endogenous brain repair mechanisms after a stroke while overcoming the limitations of cell-based therapy [30]. In order to better understand these mechanisms, this study also analyzed the brain tissue levels and in vitro differentiation of repair-related markers. In the in vivo study, the levels of MOG and MBP, markers related to oligodendrocyte differentiation and myelin fiber maturation, respectively, were higher in all the treated groups compared to the controls. These results agree with previous studies in which oligodendrocyte progenitor cells and mature oligodendrocytes were significantly increased after EV treatment [13, 31]. These results also correlate with those obtained in vitro, where 50 μg, 100 μg, and 200 μg of EVs promoted an increase of proliferation markers of OPC to oligodendrocyte (MOG) subjected to OGD conditions, with EVs playing an essential role in oligodendrocyte maturation and myelination. On the other hand, MAP-2 is a mature-specific marker and plays a role in the stability of axons and neuronal cell bodies through the differentiation process. In this study, there was also a significant increase in neural differentiation levels of MAP-2 marker in the neurons treated with at least 50 μg of EVs compared with cells receiving the lower doses. These results suggest that lower doses of EVs are not sufficient to reach the later stages of neuronal maturation.

Synaptophysin is an indicator of synaptic plasticity and synaptogenesis. In this study, levels of SYP were significantly higher in intermediate dose group compared to the control group. These data suggest that EV treatment increases neurite remodeling and synaptic plasticity, as has previously been published [14].

In response to a stroke, astrocytes are activated and form a glial scar, which protects the healthy tissue from cascading uncontrolled tissue damage. GFAP has been suggested to be a specific marker of astrocyte activation which may play controversial roles in cerebral damage. For example, astrocyte over-activation might inhibit axonal regeneration [32]. In this study, EV treatment reduced astrogliosis, as evidenced by decreased GFAP expression.

BDNF is a member of the neurotrophin family of trophic factors, which mediates activities related to neuronal proliferation, survival, differentiation, and plasticity in many neuronal populations of the central and peripheral nervous system [33]. In our study, only low dose was associated with a significant increase in BDNF expression compared to the control group. The findings suggest that low doses of EVs have great potential as a regenerative therapy for stroke.

## Conclusions

This study helped to identify 50 μg of MSC-derived EVs as the optimal effective dose to enhance brain protection, repair, and recovery after a subcortical ischemic experimental stroke. This dose showed a clear effect of improving functional outcomes associated with increased cell proliferation and decreased cell death, as well as restoration of fiber tract connectivity, increased numbers of oligodendrocyte markers, and remyelination in an experimental animal model of subcortical infarction. The in vitro study confirmed the results obtained in the in vivo study and showed that doses below 50 μg of EVs are not sufficient to achieve all these benefits.

## Supplementary information

**Additional file 1: Figure S1.** Representative immunofluorescence images of the absence of HIF-1 under conditions of normoxia and its presence under OGD conditions.

## Data Availability

The original data are available from the corresponding author on request.

## Abbreviations

A.U: Arbitrary units
BBB: Blood-brain barrier
BDNF: Brain-derived neurotrophic factor
bFGF: Basic fibroblast growth factor
DAPI: 4′,6-Diamidino-2-phenylindole
DCX: Doublecortin
DTI: Diffusion tensor imaging
EPI: Echo planar imaging
EVs: Extracellular vesicles
GFAP: Glial fibrillary acidic protein
HIF-1: Hypoxia-inducible factor
MAP-2: Microtubule associated protein 2
MBP: Myelin basic protein
MOG: Myelin-oligodendrocyte glycoprotein
MRI: Magnetic resonance imaging
MSC: Mesenchymal stem cell
OGD: Oxygen-glucose deprivation
PBS: Phosphate-buffer saline
PDGF: Platelet-derived growth factor
SD: Standard deviation
SYP: Synaptophysin
tPA: Tissue plasminogen activator

## References

[1] World Health Organization (WHO) (2014). Neurological disorders associated with malnutrition. Neurol. Disord. Public Heal. Challenges.

[2] Alonso de Leciñana M, Gutiérrez-Fernández M, Romano M, Cantú-Brito C, Arauz A, Olmos LE (2014). Strategies to improve recovery in acute ischemic stroke patients: Iberoamerican Stroke Group Consensus. Int J Stroke.

[3] Albers GW, Marks P, Kemp S (2018). DEFUSE 3 investigators. Thrombectomy for stroke at 6 to 16 hours with selection by perfusion imaging. N Engl J Med.

[4] Nogueira RG, Jadhav AP, Haussen DC, Bonafe A, Budzik RF, Bhuva P (2018). Thrombectomy 6 to 24 hours after stroke with a mismatch between deficit and infarct. N Engl J Med.

[5] Leu S, Lin YC, Yuen CM, Yen CH, Kao YH, Sun CK, Yip HK (2010). Adipose-derived mesenchymal stem cells markedly attenuate brain infarct size and improve neurological function in rats. J Transl Med.

[6] Chopp M, Li Y (2002). Treatment of neural injury with marrow stromal cells. Lancet Neurol.

[7] Otero-Ortega L, Gutiérrez-Fernández M, Ramos-Cejudo J, Rodríguez-Frutos B, Fuentes B, Sobrino (2015). White matter injury restoration after stem cell administration in subcortical ischemic stroke. Stem Cell Res Ther.

[8] Kalra H, Drummen GPC, Mathivanan S (2016). Focus on extracellular vesicles: introducing the next small big thing. Int J Mol Sci.

[9] Théry C, Zitvogel L, Amigorena S (2002). Exosome: composition, biogenesis and function. Nat Rev Immunol.

[10] Chen J, Chopp M (2018). Exosome therapy for stroke. Stroke.

[11] Vizoso FJ, Eiro N, Cid S, Schneider J, Perez-Fernandez R (2017). Mesenchymal stem cell secretome: toward cell-free therapeutic strategies in regenerative medicine. Int J Mol Sci.

[12] Doeppner TR, Herz J, Görgens A, Schlechter J, Ludwig AK, Radtke S (2015). Extracellular vesicles improve post-stroke neuroregeneration and prevent post-ischemic immunosuppression. Stem Cells Transl Med.

[13] Otero-Ortega L, Laso-García F, Gómez-de Frutos MD, Rodríguez-Frutos B, Pascual-Guerra J, Fuentes B (2017). White matter repair after extracellular vesicles administration in an experimental animal model of subcortical stroke. Sci Rep.

[14] Xin H, Li Y, Cui Y, Yang JJ, Zhang ZG, Chopp M (2013). Systemic administration of exosomes released from mesenchymal stromal cells promote functional recovery and neurovascular plasticity after stroke in rats. J Cereb Blood Flow Metab.

[15] Chen KH, Chen CH, Wallace CG, Yuen CM, Kao GS, Chen YL (2016). Intravenous administration of xenogenic adipose-derived mesenchymal stem cells (ADMSC) and ADMSC-derived exosomes markedly reduced brain infarct volume and preserved neurological function in rat after acute ischemic stroke. Oncotarget.

[16] Otero-Ortega L, Gómez de Frutos MC, Laso-García F, Rodríguez-Frutos B, Medina-Gutiérrez E, López JA (2018). Exosomes promote restoration after an experimental animal model of intracerebral haemorrhage. J Cereb Blood Flow Metab.

[17] Otero-Ortega L, Laso-García F, Gómez-de Frutos M, Fuentes B, Diekhorst L, Díez-Tejedor E, Gutiérrez-Fernández M (2019). Role of exosomes as a treatment and potential biomarker for stroke. Transl Stroke Res.

[18] Moniche F, Rosado-de-Castro PH, Escudero I, Zapata E, de la Torre Laviana FJ, Mendez-Otero R (2016). Increasing dose of autologous bone marrow mononuclear cells transplantation is related to stroke outcome: results from a pooled analysis of two clinical trials. Stem Cells Int.

[19] Stroke Therapy Academic Industry Roundtable (STAIR) (1999). Recommendations for standards regarding preclinical neuroprotective and restorative drug development. Stroke.

[20] Fisher M, Feuerstein G, Howells DW, Hurn PD, Kent TA, Savitz SI, Lo EH, STAIR Group (2009). Update of the stroke therapy academic industry roundtable preclinical recommendations. Stroke.

[21] Kilkenny C, Browne W, Cuthill IC, Emerson M, Altman DG (2011). National Centre for the replacement, refinement and reduction of amimals in research. Animal research: reporting in vivo experiments--the ARRIVE guidelines. J Cereb Blood Flow Metab.

[22] Prescott MJ, Lidster K (2017). Improving quality of science through better animal welfare: the NC3Rs strategy. Lab Anim.

[23] Britton GL, Kim H, Kee PH, Aronowski J, Holland CK, McPherson DD, Huang SL (2010). In vivo therapeutic gas delivery for neuroprotection with echogenic liposomes. Circulation.

[24] Rodríguez-Frutos B, Otero-Ortega L, Ramos-Cejudo J, Martínez-Sánchez P, Barahona-Sanz I, Navarro-Hernanz T (2016). Enhanced brain-derived neurotrophic factor delivery by ultrasound and microbubbles promotes white matter repair after stroke. Biomaterials.

[25] Rogers DC, Campbell CA, Stretton JL, Mackay KB (1997). Correlation between motor impairment and infarct volume after permanent and transient middle cerebral artery occlusion in the rat. Stroke.

[26] Xin H, Li Y, Liu Z, Wang X, Shang X, Cui Y (2013). MiR-133b promotes neural plasticity and functional recovery after treatment of stroke with multipotent mesenchymal stromal cells in rats via transfer of exosomes-enriched extracellular particles. Stem Cells.

[27] Dijkhuizen RM, Ren J, Mandeville JB, Wu O, Ozdag FM, Moskowitz MA (2001). Functional magnetic resonance imaging of reorganization in rat brain after stroke. Proc Natl Acad Sci U S A.

[28] Kawamata T, Dietrich WD, Schallert T, Gotts JE, Cocke RR, Benowitz LI (1997). Intracisternal basic fibroblast growth factor enhances functional recovery and up-regulates the expression of a molecular marker of neuronal sprouting following focal cerebral infarction. Proc Natl Acad Sci U S A.

[29] Liu HS, Shen H, Harvey BK, Castillo P, Lu H, Yang Y (2011). Post-treatment with amphetamine enhances reinnervation of the ipsilateral side cortex in stroke rats. Neuroimage.

[30] Venkat P, Chen J, Chopp M (2018). Exosome-mediated amplification of endogenous brain repair mechanisms and brain and systemic organ interaction in modulating neurological outcome after stroke. J Cereb Blood Flow Metab.

[31] Xin H, Katakowski M, Wang F, Qian JY, Liu XS, Ali MM (2017). MicroRNA cluster miR-17-92 cluster in exosomes enhance neuroplasticity and functional recovery after stroke in rats. Stroke.

[32] Pan Q, He C, Liu H, Liao X, Dai B, Chen Y (2016). Microvascular endothelial cells-derived microvesicles imply in ischemic stroke by modulating astrocyte and blood brain barrier function and cerebral blood flow. Mol Brain.

[33] Reichardt LF (2006). Neurotrophin-regulated signaling pathways. Philosophical Transactions of the Royal Society. Biological Sciences.

