# Supplementary material for: Low dose of extracellular vesicles identified that promote recovery after ischemic stroke
Source: Stem Cell Res Ther. 2020 Feb 19;11:70. doi: 10.1186/s13287-020-01601-1 (PMC7029550; doi:10.1186/s13287-020-01601-1)
Supplement: Supplementary file 1 — Additional file 1: Figure S1. Representative immunofluorescence images of the absence of HIF-1 under conditions of normoxia and its presence under OGD conditions. [file 13287_2020_1601_MOESM1_ESM.docx]

**Low dose of extracellular vesicles identified that promote recovery after ischemic stroke**

**Authors:** Laura Otero-Ortega^1†^, Fernando Laso-García^1†^, Mari Carmen Gómez-de Frutos^1†^, Luke Diekhorst^1^, Arturo Martínez-Arroyo^1^, Elisa Alonso-López^1^, María Laura García-Bermejo^2^, Macarena Rodríguez-Serrano^2^, Mercedes Arrúe-Gonzalo^1^, Exuperio Díez-Tejedor^1†^, Blanca Fuentes^1†*^, María Gutiérrez-Fernández^1†*^

^1^Neuroscience and Cerebrovascular Research Laboratory, Department of Neurology and Stroke Center, La Paz University Hospital, Hospital La Paz Institute for Health Research (IdiPAZ), Autonomous University of Madrid, Madrid, Spain

^2^Biomarkers and Therapeutic Targets Unit, Instituto Ramón y Cajal de investigación Sanitaria (IRYCIS)

^†^These authors contributed equally to this work

^*^corresponding author

**
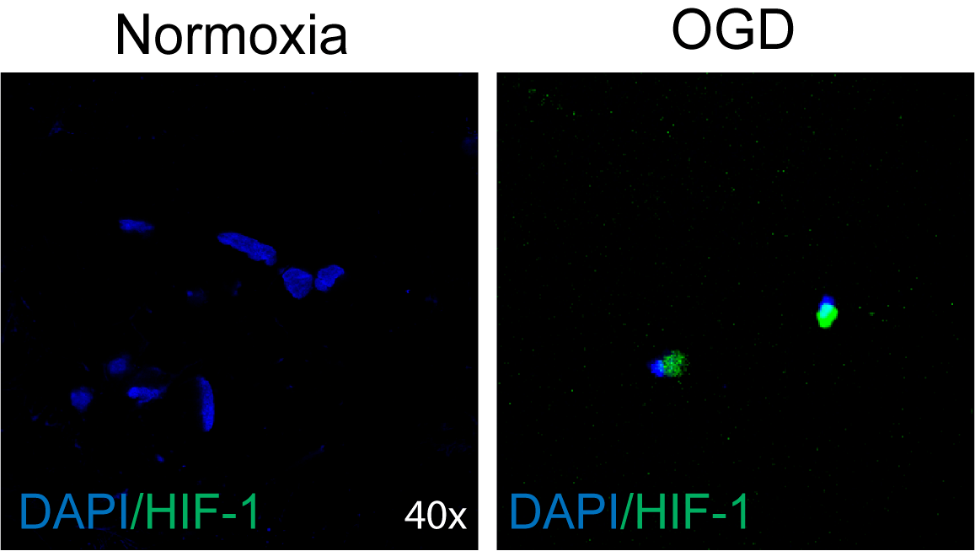
**

**Fig. Suppl 1** Representative immunofluorescence images of the absence of HIF-1 under conditions of normoxia and its presence under OGD conditions.
